# NLRP3 activation in macrophages promotes acute intestinal injury in neonatal necrotizing enterocolitis

**DOI:** 10.1007/s12519-023-00727-5

**Published:** 2023-06-30

**Authors:** Bo Shi, Cheng-Jie Lyu, Zhen-Kai Le, Hao-Sen Ji, Yi Xiao, Yuan-Yuan Zhang, Shou-Jiang Huang, Lin-Jun Yu, Qiang Shu, Jin-Fa Tou, Deng-Ming Lai

**Affiliations:** 1 Department of Neonatal Surgery, Children’s Hospital, Zhejiang University School of Medicine, National Clinical Research Center for Child Health, Hangzhou, 310052 China; 2 Department of Pulmonology, Children’s Hospital, Zhejiang University School of Medicine, National Clinical Research Center for Child Health, Hangzhou, 310052 China; 3 Department of Thoracic and Cardiovascular Surgery, Children’s Hospital, Zhejiang University School of Medicine, National Clinical Research Center for Child Health, Hangzhou, 310052 China

**Keywords:** Caspase-1, Interleukin-1β, Macrophage, Necrotizing enterocolitis, NLRP3

## Abstract

**Background:**

Macrophages are involved in various immune inflammatory disease conditions. This study aimed to investigate the role and mechanism of macrophages in regulating acute intestinal injury in neonatal necrotizing enterocolitis (NEC).

**Methods:**

CD68, nucleotide-binding oligomerization domain, leucine-rich repeat, and pyrin domain-containing 3 (NLRP3), cysteine aspartate-specific protease-1 (caspase-1), and interleukin-1β (IL-1β) in paraffin sections of intestinal tissues from NEC and control patients were detected with immunohistochemistry, immunofluorescence, and western blot. Hypertonic pet milk, hypoxia and cold stimulation were used to establish a mouse (wild type and *Nlrp3*^*−/−*^) model of NEC. The mouse macrophage (RAW 264.7) and rat intestinal epithelial cell-6 lines were also cultured followed by various treatments. Macrophages, intestinal epithelial cell injuries, and IL-1β release were determined.

**Results:**

Compared to the gut “healthy” patients, the intestinal lamina propria of NEC patients had high macrophage infiltration and high NLRP3, caspase-1, and IL-1β levels. Furthermore, in vivo, the survival rate of *Nlrp3*^*−/−*^ NEC mice was dramatically improved, the proportion of intestinal macrophages was reduced, and intestinal injury was decreased compared to those of wild-type NEC mice. NLRP3, caspase-1, and IL-1β derived from macrophages or supernatant from cocultures of macrophages and intestinal epithelial cells also caused intestinal epithelial cell injuries.

**Conclusions:**

Macrophage activation may be essential for NEC development. NLRP3/caspase-1/IL-1β cellular signals derived from macrophages may be the underlying mechanism of NEC development, and all these may be therapeutic targets for developing treatments for NEC.

**Supplementary Information:**

The online version contains supplementary material available at 10.1007/s12519-023-00727-5.

## Introduction

Neonatal necrotizing enterocolitis (NEC) is one of the most common and severe bowel disease emergencies in neonates, especially preterm infants [1, 2]. The overall morbidity and mortality of patients with NEC are high because most patients are preterm and low birth weight vulnerable infants [3, 4, 5, 6, 7, 8]. These infants are susceptible to inflammation and biological abnormalities due to immature intestinal development, inadequate intestinal flora disorder, and inadequate feeding methods (hypertonic milk and formula milk), resulting in the development of NEC. Human milk feeding can ease inflammation and biological dysfunction and minimize the incidence of NEC [9]. Worrisomely, the complications of NEC, such as intestinal necrosis, septic shock, short bowel syndrome, nervous system damage, and growth retardation, seriously affect patient survival and quality of life. At a cellular level, intestinal epithelial barrier integrity loss and intestinal epithelial cell injury or death are the main pathological changes in this disease [10, 11]. Consequently, microbial pathogen invasion triggers the innate immune response via pathogen-associated molecular patterns and/or danger-associated molecular patterns [12, 13]. Macrophages are a key component of the innate immune system, which helps protect the body from disease by destroying pathogens, secreting inflammatory mediators and inducing cell death [14, 15]. Intestinal-resident macrophages, in contrast to monocytes and other tissue-resident macrophages, normally exhibit low inflammatory reactivity and high phagocytic activity [16]. Once the microenvironment is disrupted, macrophages undergo a variety of alterations and participate in different immune inflammatory responses, which can influence the onset and progression of disease [16, 17, 18]. Nonetheless, the interaction and mechanism between macrophages and intestinal epithelial cells are still not fully understood.

The nucleotide-binding oligomerization domain, leucine-rich repeat, and pyrin domain-containing 3 (NLRP3) inflammasome is an essential mediator of innate immunity in response to malignancies, nerve injury, inflammatory illnesses, and metabolic abnormalities [19, 20]. As a key component of macrophages, NLRP3 governs the inflammatory immune response and controls pyroptosis [21, 22]. Caspase-1 is a crucial NLRP3 downstream regulator that controls macrophage pyroptosis [23, 24]. NLRP3 released from dead macrophages promotes the maturation of pro-interleukin (IL)-1β and pro-interleukin-18 into active forms mediating disease progression [25]. Indeed, it has been reported that activation of the NLRP3 inflammasome influences the occurrence and progression of NEC [26]. NLRP3 was found to be considerably activated in the intestinal tissue of NEC patients, and decreasing NLRP3 activity reduced intestinal inflammation and increased survival, as shown in an animal model of NEC [27, 28, 29]. Nevertheless, it is unknown whether inflammasome activation in intestinal macrophages plays a key role in intestinal epithelial injury or death during NEC.

This study aims to investigate the role of NLRP3 activation in macrophages in NEC intestinal injury and the underlying molecular mechanisms and to determine the potential protective effect of inhibiting NLRP3 activation on intestinal epithelial cell injury. The ultimate aim of our study may provide a foundation for developing future NEC treatment strategies.

## Methods

### Human samples

After obtaining ethics approval from the Ethics Committee of Children’s Hospital, Zhejiang University School of Medicine (2019-IRB-088) and written informed consent from the patient’s parents, intestinal samples from eighteen NEC patients (median age: 24 days, median weight: 1510 g) and four patients with intestinal atresia (IA) (median age: 2.5 days, median weight: 3265 g) were collected during surgery between December 2021 and June 2022 in the Department of Neonatal Surgery, Children’s Hospital, Zhejiang University School of Medicine (Table 1).

**Table 1:** Characteristics of neonates in necrotizing enterocolitis group and control group

Characteristics	NEC (*n* = 18)	Control (*n* = 4)
Birth weight (g), median (range)	1510 (1061–1865)	3265 (3098–3482)
Gestational week (wk), mean ± SD	30.00 ± 4.04	37.75 ± 0.96
Premature, *n* (%)		
No	2 (9.1)	4 (18.2)
Yes	16 (72.7)	0 (0.0)
Gender, *n* (%)		
Male	10 (45.5)	3 (13.6)
Female	8 (36.4)	1 (4.5)
Birth age (d)	24.0 (10.2–35.8)	2.5 (1.5–3.5)

*NEC* necrotizing enterocolitis, *SD* standard deviation

### Mice and necrotizing enterocolitis induction

C57BL/6 J mice were purchased from Shanghai Slac Laboratory Animal Company (Shanghai, China). *Nlrp3*^*−/−*^ mice were donated by Professor Di Wang at the Institute of Immunology, Zhejiang University School of Medicine. Mice (5 days old) weighing about 2.0 g were used to establish the model of NEC as reported previously with some modifications [30]. Briefly, neonatal mice were fed 50 μL of 30% Esbilac formula (Pet-Ag, New Hampshire, IL, USA) by gavage every 4 h for 96 h through a 1.9-French angio-catheter placed into the esophagus under direct vision. They were then subjected to hypoxia (99.9% N_2_ for 90 s) in a hypoxic chamber (Aipuins, Hangzhou, China) followed by cold stress (4 °C for 10 min) twice daily for 4 days [30]. Terminal ileum samples were harvested under euthanasia at the end of the experiments for subsequent analysis. Pups were randomized into the following groups: (1) wild type (WT)-control (breastfed, *n* = 15); (2) WT-NEC (*n* = 15). Similarly, the pups of the *Nlrp3*^*−/−*^ mice were randomized into the following groups: (1) *Nlrp3*^*−/−*^-control (breastfed, *n* = 15); (2) *Nlrp3*^*−/−*^-NEC (*n* = 15). The experimental protocol followed the guidelines for the ethical treatment of experimental animals and was approved by the Institutional Animal Care and Use Committee of the Zhejiang University School of Medicine.

### Hematoxylin and eosin staining

Hematoxylin and eosin (HE) staining was carried out to assess the degree of intestinal lesions. All obtained intestinal samples were fixed with 4% paraformaldehyde for 24–48 h, sliced after paraffin embedding, stained with HE, and examined using an Olympus light microscope. Histological changes in the terminal ileum were used to diagnose NEC using a previously established scoring system [31]. The grading scheme is as follows: normal ileum graded as 0; mild injury to the villus tip graded as 1; partial villus loss graded as 2; severe submucosal injury graded as 3, and complete necrosis as graded 4. A grade of 2 or higher was considered to indicate the presence of NEC [31].

### Isolation of mouse intestinal macrophages

Small intestine macrophages were isolated using a previously published protocol with minor modifications [32]. Briefly, the small intestine from neonatal mice was removed and cut longitudinally, excluding the duodenum. The tissue fragments were transferred to a solution (1 mM dithiothreitol + 5 mM ethylene diamine tetraacetic acid) and incubated in a shaker at 37 °C for 30 min after being washed in phosphate-buffered saline (PBS). The tissues were then transferred into solution [Dulbecco’s modified Eagle’s medium (DMEM) + 5% bovine serum albumin (BSA) + 75 µg/mL Liberase TM], and the above procedures were repeated. After filtering, the cells were washed 2–3 times with PBS and stained with fixable viability stain 510, F4/80, CD11b, and CD45 antibodies for flow cytometry analysis.

### Cell culture

Mouse macrophage (RAW 264.7) and rat intestinal epithelial cell (IEC-6) lines were obtained from the American Type Culture Collection (ATCC) and Cell Bank in Shanghai, China. All cells were cultured in DMEM supplemented with 10% fetal bovine serum (Ausgenex, Australia) and 1% penicillin/streptomycin (Sangon Biotech, Shanghai, China) at 37 °C and 5% CO_2_. RAW 264.7 cells were seeded at 5 × 10^5^ cells/mL in 6-well plates or 1 × 10^5^ cells/mL in 24-well plates, and IEC-6 cells were seeded at 2 × 10^5^ cells/mL in 6-well plates. After 24 h, IEC-6 cells were treated with 1 µg/mL lipopolysaccharide (LPS; L2654, Sigma‒Aldrich, MO, USA). RAW 264.7 cells were treated with 1 µg/mL LPS, 1 µg/mL LPS and 1 µM MCC950 (a specific inhibitor of the NLRP3 inflammasome; CAS 256373-96-3, Sigma‒Aldrich, MO, USA) or 1 µg/mL LPS and 10 µM Z-YVAD-FMK (218746, caspase-1 inhibitor VI, Sigma‒Aldrich, MO, USA) [33].

After 24 h of LPS stimulation, the supernatant of RAW 264.7 cells was collected by centrifugation at 8000 rpm for 15 min and used in subsequent experiments. In addition, in the IL-1β neutralization experiment, 0.5 μg/mL IL-1β neutralizing antibody (AF-401-NA, R&D Systems Inc., Minnesota, USA) was added to the supernatant of LPS-stimulated RAW 264.7 cells in advance and incubated at 4 °C for 24 h. Then, the supernatant was collected by centrifugation at 8000 rpm for 15 min and used in subsequent experiments. The supernatant and treated cells were collected for subsequent analysis.

### Flow cytometry

Treated macrophages and intestinal epithelial cells were double-stained with annexin V-fluorescein isothiocyanate and propidium iodide (566547, FITC Apoptosis Detection Kit I; BD, CA, USA) to assess cell death. Activated caspase-1 was visualized with a FAM-FLICA caspase-1 assay kit using the FAM-YVAD-FMK inhibitor probe (ImmunoChemistry Technologies, MN, USA) according to the manufacturer’s guidelines. The fluorescence intensity of caspase-1 in the different treatment groups was analyzed. Intestinal lamina propria macrophages were stained with antibodies specific for CD68 (sc-20060, Santa Cruz, USA), F4/80 (565411, BD, USA), CD11b (53-0112-82 and 557743, Invitrogen/BD, USA), CD45 (557659 and 560779, BD, USA), and fixable viability stain 510 (564406, BD, USA). Quantification was then performed using a FACSLyric™ flow cytometer (663518, BD, NJ, USA) and analyzed using FlowJo software.

### Western blot

Total protein was extracted from macrophages with different treatments or tissues using radioimmunoprecipitation assay lysis buffer (Boster, Wuhan, China), and the concentration of proteins was determined by a BCA protein assay kit (Meilunbio, Dalian, China). Cell lysates were resolved by SDS‒PAGE, and polyvinylidene difluoride (Immobilon-P, Merck, USA) membranes were probed with anti-NLRP3 (AG-20B-0014, 1:1000, Adipogen, South Korea), anti-caspase-1/P20/P10 (22915-1-AP, 1:1000, Proteintech, Wuhan, China), or anti-glyceraldehyde-3-phosphate dehydrogenase (60004-1-1G, 1:5000, Proteintech, Wuhan, China) antibodies and then developed by chemiluminescence (Thermo Fisher Scientific, MA, USA). Relative protein levels were quantified with the GeneSys Chemi imaging system (Syngene G:BOX, USA) and ImageJ (National Institutes of Health, MD, USA).

### Immunohistochemical staining

Immunohistochemical staining was performed as previously described [27]. The paraffin sections were incubated overnight at 4 °C with primary antibodies, anti-CD68 (sc-20060, 1:200, Santa Cruz, USA) or anti-IL-1β (ab9722, 1:200, Abcam, Cambridge, UK), followed by incubation with horseradish peroxidase-coupled secondary antibodies at room temperature for 1 h. Finally, DAB solution (Sangon Biotech, Shanghai, China) and hematoxylin were used for staining. High-resolution digital images were captured with a high-speed digital slide scanner (3DHistech, Hungary).

### Immunofluorescence staining

After the tissues were blocked with 5% BSA for 1 h, they were incubated overnight with anti-CD68 and anti-NLRP3 antibodies or anti-CD68 and anti-caspase-1 (1:200 in 5% BSA) followed by staining with Alexa Fluor 488-labeled and 594-labeled secondary antibodies (111-545-144 and 115-585-146, Jackson ImmunoResearch, PA, USA). The nuclei were counterstained with 4,6-diamidino-2-phenylindole (E607303, BBI, Shanghai, China) and then viewed under a Zeiss microscope (Carl Zeiss, Jena, Germany).

### Enzyme-linked immunosorbent assay

The amount of IL-1β protein in the culture supernatants was measured using mouse-specific IL-1β enzyme-linked immunosorbent assay ELISA kits (abs520001, Absin, Shanghai, China) according to the manufacturer’s instructions.

### Statistical analysis

All experiments were performed independently at least three times. Descriptive data were expressed as the median (arrange), or measurement data were expressed as the mean ± standard error of the mean. Data were then analyzed by Mann–Whitney *U* test, two-tailed unpaired Student’s *t* tests or one-way analysis of variance followed by post hoc test with R (version 4.2.1; statistical analysis and visualization) where appropriate. A value of *P* < 0.05 was considered to be statistically significant.

## Results

### Macrophages accumulate in the intestinal tissues of patients with NEC and induce intestinal epithelial cell injury

**Fig. 1:**
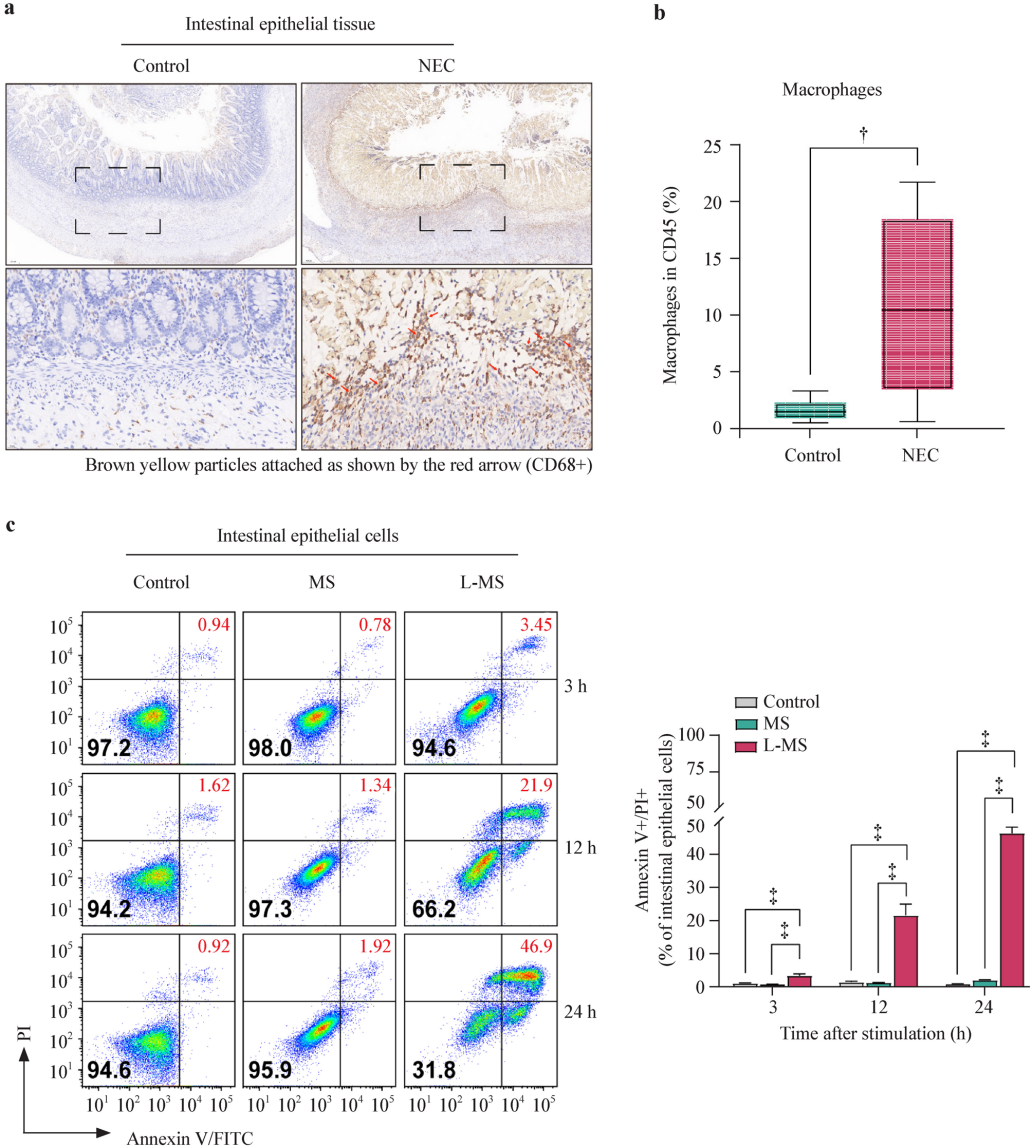
Macrophages accumulate in the intestinal tissues of patients with NEC and induce intestinal epithelial cell injury. **a** Immunohistochemistry of macrophages (marked by CD68) in intestinal tissue of intestinal atresia (control) or NEC patients; **b** the intestinal macrophage percentages in CD45^+^ cells of control or NEC intestine tissues; **c** representative flow cytometry plots show annexin V/PI staining of intestinal epithelial cells stimulated with MS or L-MS for 3, 12, and 24 h. The bar graph shows the percentages of annexin V/PI double-stained intestinal epithelial cells. Data are representative of three independent in vitro experiments. Data are presented as the mean ± SEM. ^*^P < 0.05, ^†^*P* < 0.01, ^‡^*P* < 0.001. *NEC* necrotizing enterocolitis, *PI* propidium iodide, *MS* macrophage supernatants, *L-MS* lipopolysaccharide-induced macrophage supernatants, *FITC* fluorescein isothiocyanate, *SEM* standard error of the mean

Compared with the intestinal tissues of the IA group, the intestinal tissue of the NEC group showed a greater loss of villus structure, with more severe submucosal damage (partial necrosis) and more inflammatory cell infiltration. Extensive CD68-positive macrophage infiltration was observed in the submucosal and muscular layers of the NEC-inflamed intestinal tissue (Fig. 1a). Flow cytometry analysis further revealed a significant increase in macrophages among NEC intestinal white blood cells, which was in line with the in vivo data (Fig. 1b).

To further explore the interaction between intestinal epithelial cells and macrophages, intestinal epithelial cells were cultured with the supernatant of macrophage culture medium stimulated with or without LPS for 3, 12, and 24 h and analyzed with flow cytometry (Supplementary Fig. 1a and 1b**)**. Flow cytometric analysis with annexin V/propidium iodide showed that injury to intestinal epithelial cells could not be observed after LPS treatment. In contrast, macrophages were damaged after LPS stimulation, and the proportion of damaged cells gradually increased with LPS stimulation. Interestingly, unstimulated macrophage supernatants failed to damage intestinal epithelial cells, and the stimulated macrophage supernatant significantly increased intestinal epithelial cell injury. The proportion of intestinal epithelial cell damage gradually increased with prolonged stimulation (Fig. 1c).

### Activated NLRP3 inflammasomes in macrophages mediate intestinal epithelial cell injury

**Fig. 2:**
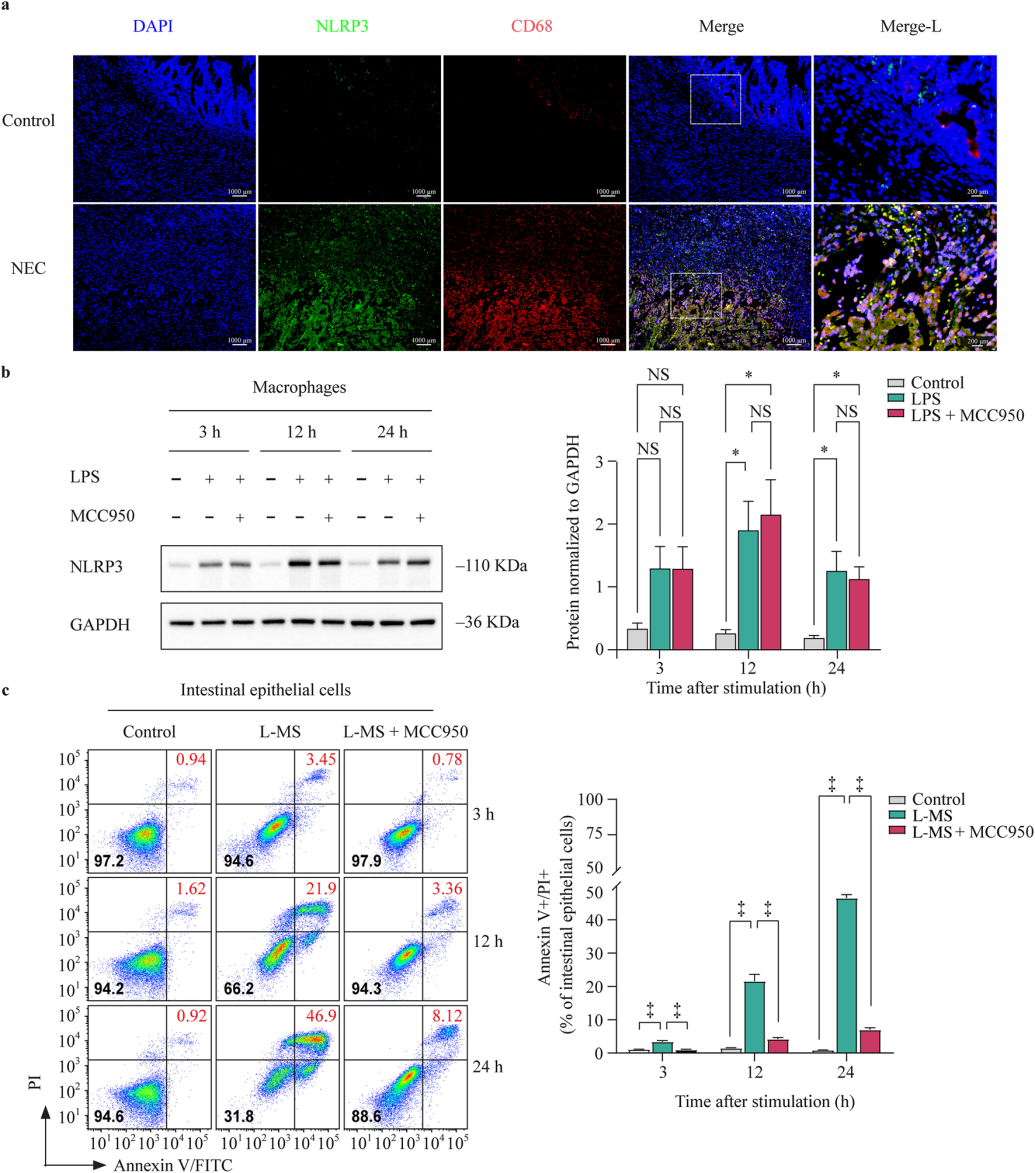
Activated NLRP3 inflammasome in macrophages induces intestinal epithelial cell injury. **a** Representative images show immunofluorescence double staining of macrophages and NLRP3. Macrophages are indicated by the colocalization of CD68 and NLRP3 in the merged images; **b** western blot analysis of NLRP3 expression in macrophages challenged with LPS (1 μg/mL) or MCC950 (NLRP3 inhibitor, 1 μM) for 3, 12, and 24 h. GAPDH was used as a loading control. The bar graph shows the relative intensity of the bands; **c** flow cytometry plots show annexin V/PI staining of intestinal epithelial cells stimulated with L-MS and (LPS + MCC950)-induced macrophage supernatants (L-MS + MCC950) for 3, 12, and 24 h. The bar graph shows the percentages of annexin V/PI double-stained intestinal epithelial cells. Data are representative of three independent in vitro experiments. Data are presented as the mean ± SEM. ^*^P < 0.05, ^†^*P* < 0.01, ^‡^*P* < 0.001. *NLRP3* nucleotide-binding oligomerization domain, leucine-rich repeat, and pyrin domain-containing 3, *DAPI* 4,6-diamidino-2-phenylindole, *LPS* lipopolysaccharide, *L-MS* lipopolysaccharide-induced macrophage supernatants, *PI* propidium iodide, *FITC* fluorescein isothiocyanate, *GAPDH* glyceraldehyde-3-phosphate dehydrogenase, *SEM* standard error of the mean, *NS* no significant difference

Macrophage infiltration and high expression of NLRP3 in mucosal and submucosal sections of the NEC-inflamed intestinal sections were readily detected (Fig. 2a). Additionally, there were more NLRP3-positive macrophages in the lamina propria of NEC intestines. Inflammation-stimulated macrophages mediate the onset and progression of NEC by activating the NLRP3 inflammasome. Furthermore, activation of the NLRP3 inflammasome was mainly from the activated macrophages in NEC samples.

The expression of NLRP3 was increased with prolonged LPS stimulation and peaked at 12 h, but MCC950 did not affect the expression of NLRP3 (Fig. 2b). The supernatant of the culture medium derived from macrophages treated with LPS or both LPS and MCC950 increased intestinal epithelial cell injury. Macrophages treated with LPS and MCC950 resulted in a lower percentage of damage than the supernatant from macrophages treated with LPS alone (Fig. 2c). These results suggest that the activation of NLRP3 is a critical step in intestinal epithelial cell injury.

### Activated caspase-1 in macrophages mediates intestinal epithelial cell injury

**Fig. 3:**
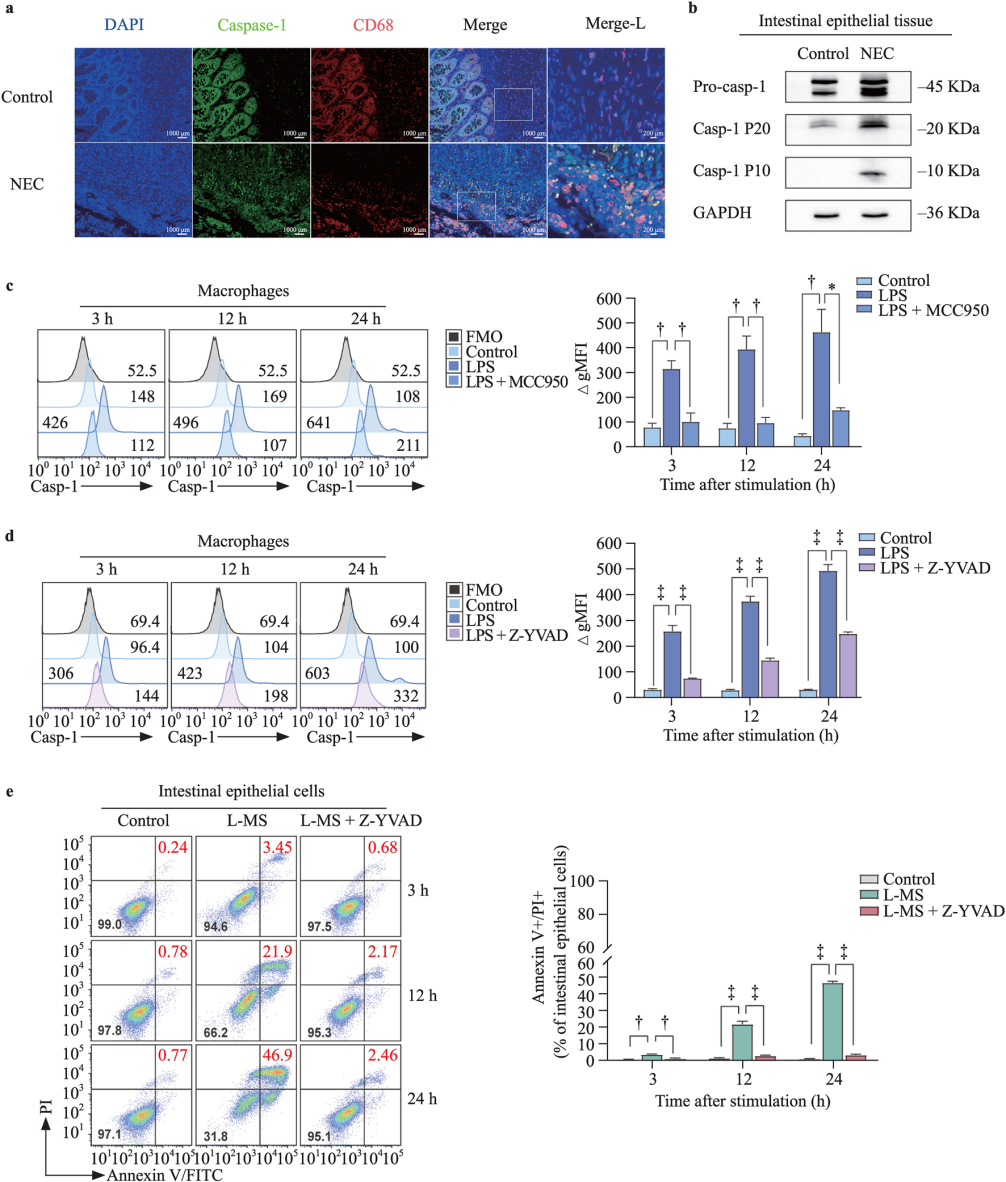
Caspase-1 is required for NLRP3 inflammasome activation in macrophage-induced intestinal epithelial cell injury. **a** Representative images show the immunofluorescent double staining of macrophages and caspase-1 in control or NEC intestine. Macrophages are indicated by the colocalization of CD68 and caspase-1 in the merged images;  **b** western blot analysis of pro-caspase-1/P20/P10 expression in control or NEC intestines. GAPDH was regarded as a loading control; **c, d** the fluorescence intensity of activated caspase-1 in different treatments of macrophages with LPS (1 μg/mL) or MCC950 (1 μM) or Z-YVAD-FMK (10 μM) was analyzed. The bar graph shows the ΔgMFI of macrophages at 3, 12, and 24 h; **e** flow cytometry plots show annexin V/PI staining of intestinal epithelial cells stimulated with L-MS and (LPS + Z-YVAD-FMK)-induced macrophage supernatants (L-MS + Z-YVAD-FMK) at 3, 12, and 24 h. The bar graph shows the percentages of annexin V/PI double-stained intestinal epithelial cells. Data are representative of three independent in vitro experiments. Data are presented as the mean ± SEM. ^*^P < 0.05, ^†^*P* < 0.01, ^‡^*P* < 0.001. *NLRP3* nucleotide-binding oligomerization domain, leucine-rich repeat, and pyrin domain-containing 3, *NEC* necrotizing enterocolitis, *GAPDH* glyceraldehyde-3-phosphate dehydrogenase, *FMO* fluorescence minus one, *LPS* lipopolysaccharide, *L-MS* lipopolysaccharide-induced macrophage supernatants, *PI* propidium iodide, *FITC* fluorescein isothiocyanate, *gMFI* geometric mean fluorescence intensity, *SEM* standard error of the mean

Compared with the control group, the NEC group showed a higher level of cleaved caspase-1 and CD68 double-positive cells in the lamina propria (Fig. 3a). Similarly, caspase-1 was activated in NEC tissue samples, and the expression of the mature subunits was significantly increased (Fig. 3b). In contrast, when MCC950 or Z-YVAD-FMK combined with LPS was used to treat macrophages, the fluorescence intensity of caspase-1 was significantly reduced (Fig. 3c and d).

We collected the cell supernatants of LPS-stimulated macrophages at 24 h and the cell supernatants of macrophages stimulated with Z-YVAD-FMK (10 μM) plus LPS. Although intestinal epithelial cells were treated with the cell supernatants for different durations, we found that the damage to intestinal epithelial cells was lower in response to Z-YVAD-FMK plus LPS-stimulated supernatants than in response to LPS-stimulated macrophage supernatants (Fig. 3e).

### Macrophage pyroptosis induced IL-1β release to mediate intestinal epithelial cell injury

**Fig. 4:**
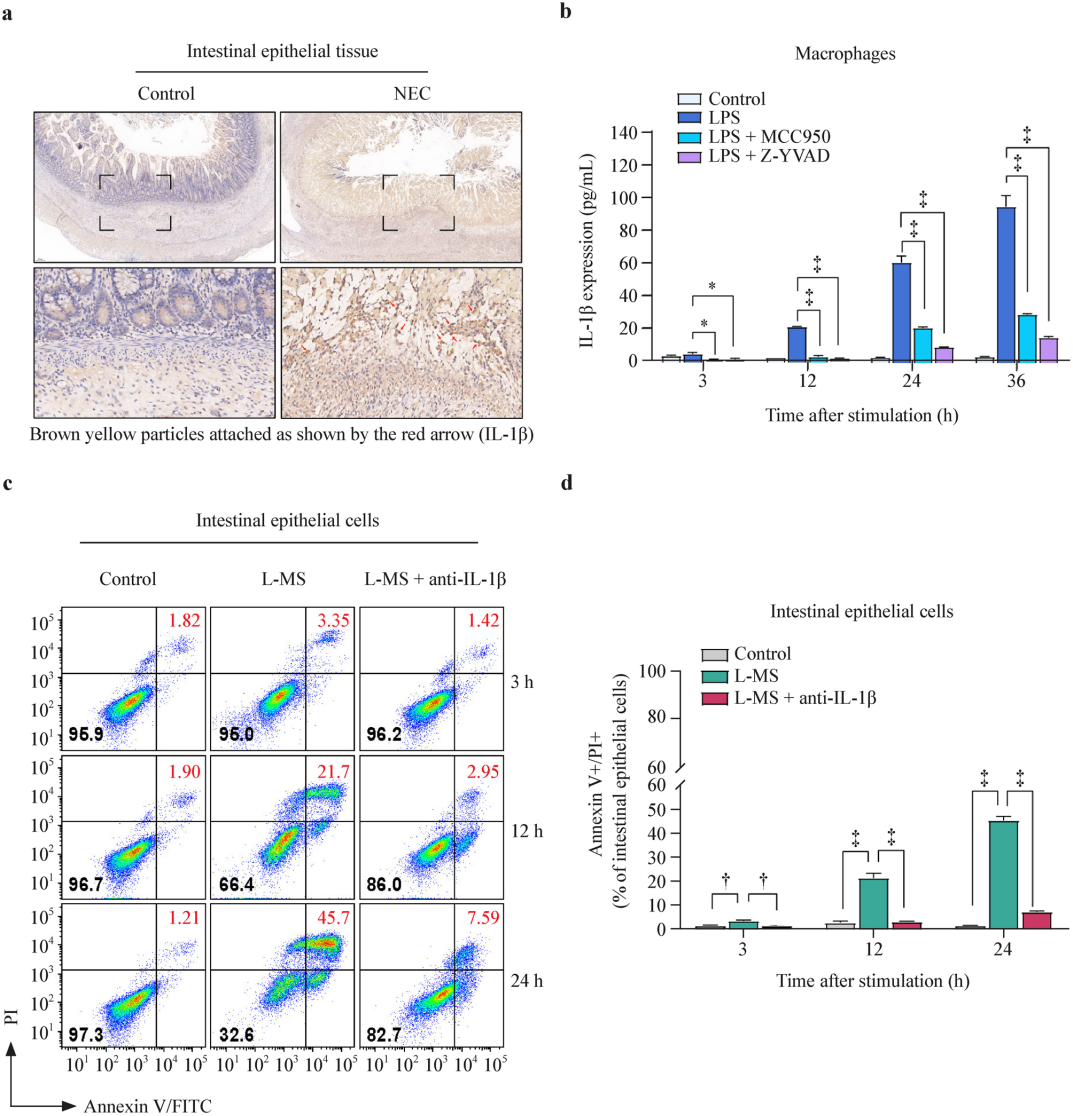
IL-1β mediates pyroptotic macrophage-induced intestinal epithelial cell injury. **a** Immunohistochemistry of IL-1β in intestine tissue of control or NEC patient; **b** the level of IL-1β in macrophages challenged with LPS (1 μg/mL) or MCC950 (1 μM) or Z-YVAD-FMK (10 μM) for 3, 12, 24, 36 h were detected with ELISA; **c, d** flow cytometry plots of annexin V/PI staining in intestinal epithelial cells stimulated with L-MS or L-MS + anti-IL-1β (IL-1β neutralizing antibody, 0.5 μg/mL) for 3, 12, 24 h. The bar graph shows the percentages of annexin V/PI double-stained intestinal epithelial cells. Data are representative of three independent in vitro experiments. Data are presented as the mean ± SEM. ^*^P < 0.05, ^†^*P* < 0.01, ^‡^*P* < 0.001. *IL* interleukin, *NEC* necrotizing enterocolitis, *LPS* lipopolysaccharide, *L-MS* lipopolysaccharide-induced macrophage supernatants, *PI* propidium iodide, *FITC* fluorescein isothiocyanate, *ELISA* enzyme-linked immunosorbent assay, *SEM* standard error of the mean

Substantial amounts of IL-1β were increased in the mucosa and submucosa of NEC intestinal tissue compared with control tissue (Fig. 4a). The content of IL-1β in the culture medium supernatant of macrophages treated with LPS or LPS combined with inhibitors was increased with LPS stimulation, but MCC950 and Z-YVAD-FMK inhibited the accumulation of IL-1β induced by LPS (Fig. 4b). The injured intestinal epithelial cells were analyzed by flow cytometry after treatment with the supernatant of LPS-stimulated macrophages for 24 h with/without 0.5 μg/mL IL-1β neutralizer. After treatment with a neutralizer, the cultured macrophages and supernatant significantly reduced the injury of intestinal epithelial cells at 12 and 24 h by more than 80% (Fig. 4c). These results indicate that IL-1β released from NLRP3 in macrophages regulates intestinal epithelial cell injury.

### NLRP3 deficiency reduced intestinal epithelial damage and improved NEC outcomes in vivo

**Fig. 5:**
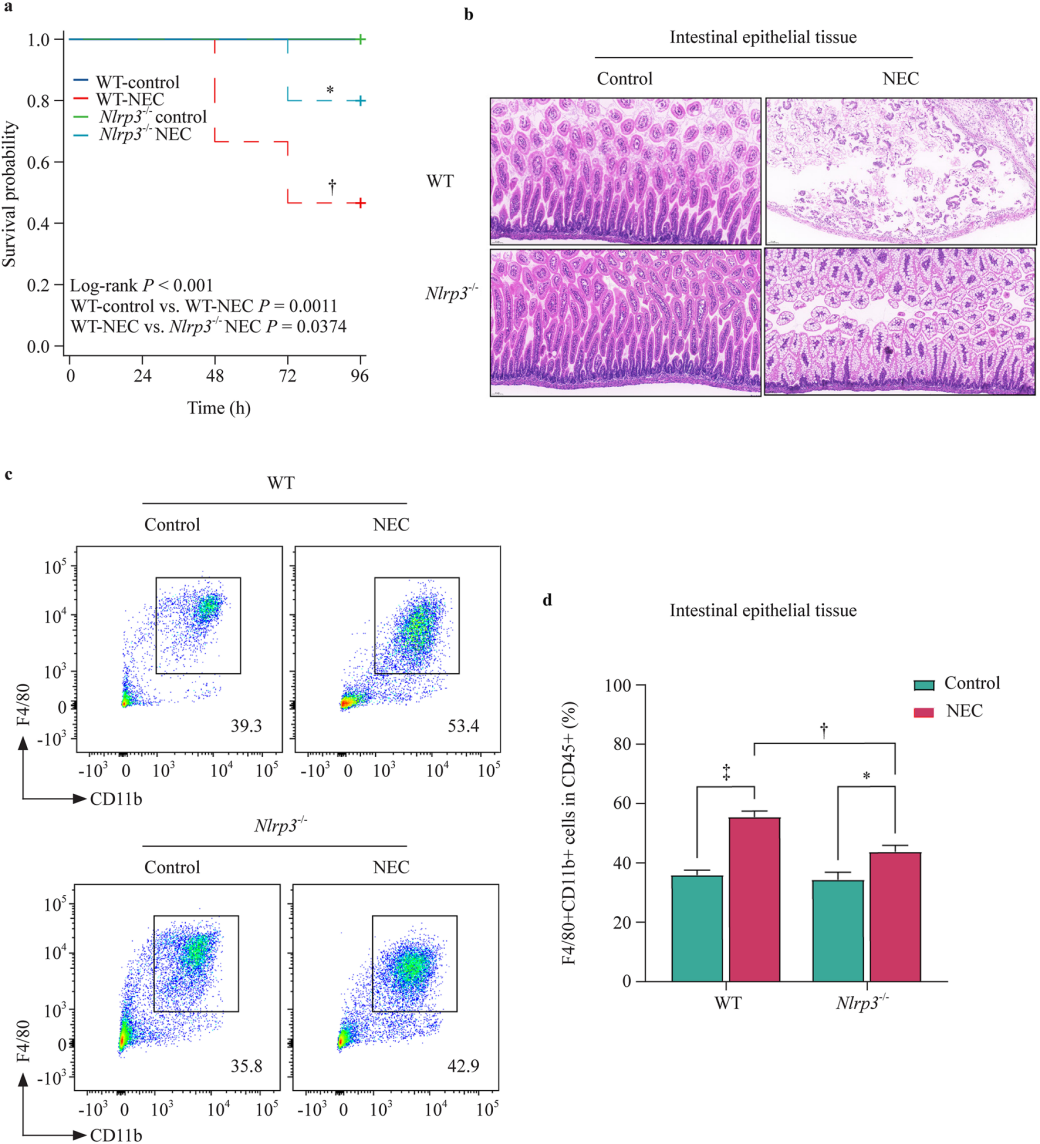
NLRP3 deficiency improves NEC mouse outcomes and decreases macrophage infiltration. Wild-type (WT) and NLRP3-deficient (*Nlrp3*^*−/−*^) mice were used to establish the model of NEC. **a** Kaplan‒Meier survival analysis of control and NEC mice (*n* = 15 per group); **b** representative pictures of hematoxylin and eosin in intestinal samples of control and NEC mice; **c, d** flow cytometry results showed the percentage of intestinal macrophages among different intestine tissues. The bar graph shows the intestinal macrophage percentages in CD45^+^ cells of control or NEC intestine tissues. Data are presented as the mean ± SEM. ^*^P < 0.05, ^†^*P* < 0.01, ^‡^*P* < 0.001. *NLRP3* nucleotide-binding oligomerization domain, leucine-rich repeat, and pyrin domain-containing 3, *NEC* necrotizing enterocolitis, *SEM* standard error of the mean

Because macrophages and NLRP3 inflammasome activation are necessary for NEC intestinal injury, we investigated whether NLRP3 deficiency prevents inflammatory injury of the intestines in an NEC mouse model. The survival rate of the WT-NEC group was 46.7%, whereas the *Nlrp3*^*−/−*^-NEC group had an 80% survival rate (*P* = 0.0374). The deletion of the *NLRP3* gene significantly improved the 96-h survival rate of a mouse NEC model (Fig. 5a). Similarly, intestinal injury in the *Nlrp3*^*−/−*^-NEC mice was greatly reduced (Fig. 5b). The ratio of intestinal macrophages to white blood cells was also significantly decreased in *Nlrp3*^*−/−*^-NEC mice compared to WT-NEC mice (Fig. 5c and d).

## Discussion

Our study with human samples in vivo and in vitro found that macrophages infiltrated the intestine and that activated NLRP3 in macrophages of the intestine triggered epithelial cell injury and death following NEC. NLRP3/caspase-1/IL-1β cellular signals derived from macrophages significantly contribute to acute intestinal injury in NEC.

To counteract the effect of the inflammatory macrophage response on tissue damage, macrophages switch to an anti-inflammatory phenotype or undergo pyroptosis to inhibit the proinflammatory response [34, 35]. However, excessive pyroptosis leads to cell death and the release of inflammatory mediators to activate and amplify inflammatory responses, thereby triggering immune disorders and excessive inflammation, resulting in a cytokine storm [36, 37]. Our study clearly demonstrated that increased NLRP3, caspase-1 and IL-1β were found in the intestinal tissues of children with NEC. Additionally, we found that the activated NLRP3 inflammasome in macrophages mediated intestinal epithelial cell injury.

Activation of NLRP3 induces the production of proinflammatory cytokines, which further recruit immune cells to infected tissues and accelerate the inflammatory response [38]. Therefore, these results may suggest that NLRP3 inflammasome inhibitors, e.g., MCC950, inhibited the activation of caspase-1, thereby inhibiting IL-1β release and subsequently reducing inflammation. MCC950 also efficiently mitigated intestinal epithelial cell injuries induced by macrophage-secreted cytokines. Additionally, the survival rate of *NLRP3*^−/−^ mice with less intestinal injury was significantly increased. Therefore, taking into account the role that activated NLRP3 plays in NEC intestinal epithelial injury, the clinical application of NLRP3 inhibitors or the identification of new targets upstream of NLRP3 for early intervention may improve NEC prognosis.

Meanwhile, our findings further indicated that activated caspase-1 and IL-1β in macrophages were associated with the development of NEC and that activated caspase-1 and IL-1β in macrophages aggravated damage to intestinal epithelial cells. As a main effector protease in the pyroptotic pathway, the processing and activation of caspase-1 promote the release of mature cytokines into the extracellular space, thereby inducing an inflammatory response [36]. Z-YVAD-FMK is capable of inhibiting caspase-1 activation, hence limiting the production of IL-1β released from activated macrophages and subsequently reducing intestinal epithelial cell injury and death. IL-1β plays a significant role in the pathophysiology of inflammatory and autoimmune illnesses, particularly intestinal inflammatory diseases. IL-1β induced increased permeability of tight junctions in intestinal epithelial cells, while intestinal barrier dysfunction was also found to be a key pathological change leading to intestinal inflammation [39]. Similarly, neutralizing IL-1β found in our study largely reduced the intestinal epithelial cell damage induced by macrophage culture supernatant. Thus, blocking caspase-1 activation reduced IL-1β or directly neutralizing or suppressing IL-1β effectively alleviated intestinal epithelial cell injury in NEC. All these factors may become further therapeutic targets to treat NEC.

Macrophage-mediated homeostasis is vitally important [40]. Macrophages in human breast milk serve a significant function in the regulation of neonatal inflammation and the stimulation of a healing response, as well as in maintaining the digestive tract balance of neonates [41]. Inflammation signifies severe imbalance, and macrophages' other major function is to reduce inflammation and facilitate repair. It reduces the generation of inflammatory cytokines and transforms macrophages into tissue repair coordinators. Nevertheless, this regulatory function of macrophages is only partially understood. Consequently, breastfeeding should be promoted aggressively for children with NEC, and the mechanism by which macrophages in breast milk repair or decrease severe intestinal injury and inflammation when NEC occurs should be investigated.

In recent years, targeting the NLRP3 inflammasome has become very promising for inflammatory disease treatment. Several inhibitors have entered into clinical studies to treat inflammatory disease conditions, including osteoarthritis, acute gout attack, heart failure, Parkinson's disease, Crohn's disease, and others [19, 42]. In addition, the treatment values of IL-1β inhibitors that impede IL-1β signal transduction or IL-1R antagonists for such conditions are also emerging [43]. Nonetheless, substantial side effects cannot be ignored, while their safety and effectiveness are also unknown, and further study is needed.

In conclusion, macrophages were found to play an essential role in the occurrence and development of NEC. The activation of NLRP3 in intestinal macrophages mediated macrophage pyroptosis and led to inflammatory mediator release, which subsequently amplified intestinal epithelial cell injury and death (Supplementary Fig. 2). Therefore, inhibiting upstream mechanisms leading to macrophage pyroptosis and subsequent downstream cell signaling pathways may reduce intestinal epithelial cell injury or death. All these factors may have therapeutic value in treating NEC.

### Supplementary Information

Below is the link to the electronic supplementary material.

Supplementary file 1 (PDF 338 KB)

## Data Availability

All data generated or analyzed during this study are included in this published article (and its supplementary information files).
