# Supplementary material for: NLRP3 activation in macrophages promotes acute intestinal injury in neonatal necrotizing enterocolitis
Source: World J Pediatr. 2023 Jun 30;20(2):153–64. doi: 10.1007/s12519-023-00727-5 (PMC10884143; doi:10.1007/s12519-023-00727-5)
Supplement: Supplementary file 1 — (PDF 338 KB) [file 12519_2023_727_MOESM1_ESM.pdf]

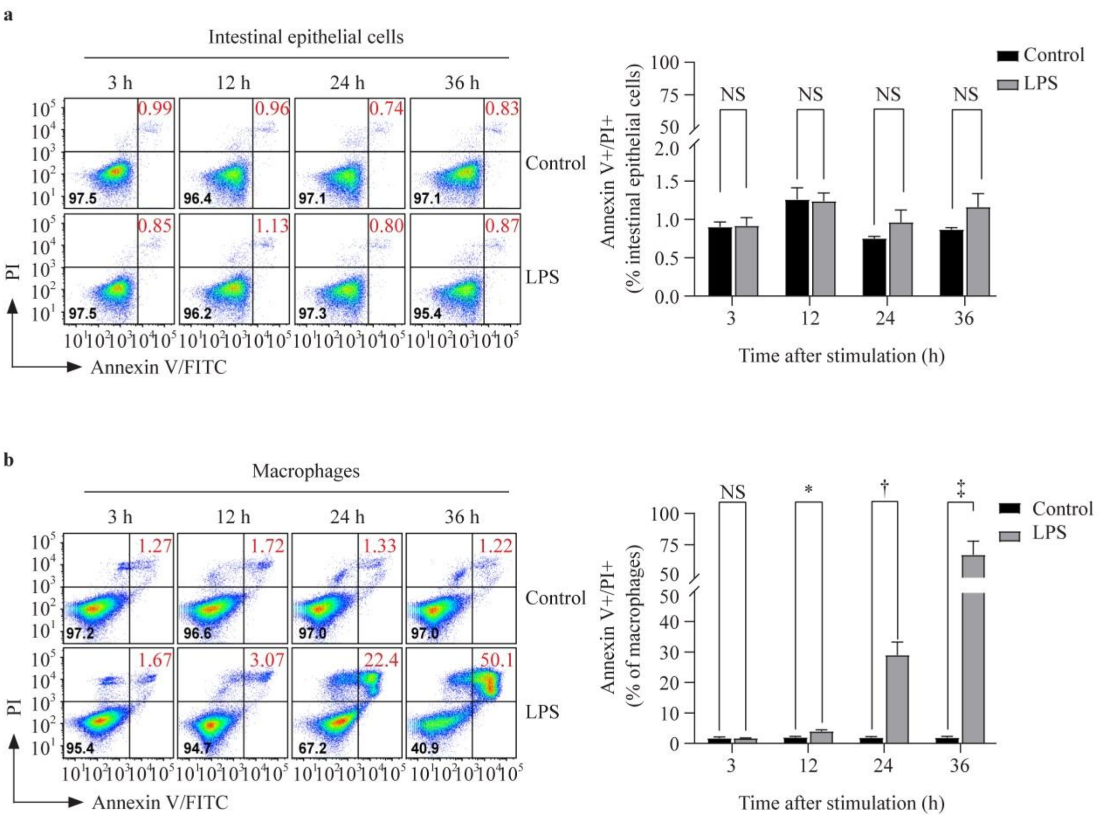

Supplementary Fig. 1 LPS induces cell death of macrophages, but not intestinal epithelial cells. **a, b** Flow cytometry plots of annexin V/PI staining in macrophages/intestinal epithelial cells stimulated with LPS (1 μg/mL) for 3 hours, 12 hours, 24 hours, and 36 hours. Bar graph shows percentages of annexin V/PI double-stained macrophages/intestinal epithelial cells. Data are representative of three independent in vitro experiments. Data are presented as mean ± SEM. $^{*}P < 0.05$, $^{\dagger}P < 0.01$, $^{\ddagger}P < 0.001$. *LPS* lipopolysaccharide, *PI* propidium iodide, *FITC* fluorescein isothiocyanate, *SEM* standard error of the mean, *NS* no significant difference

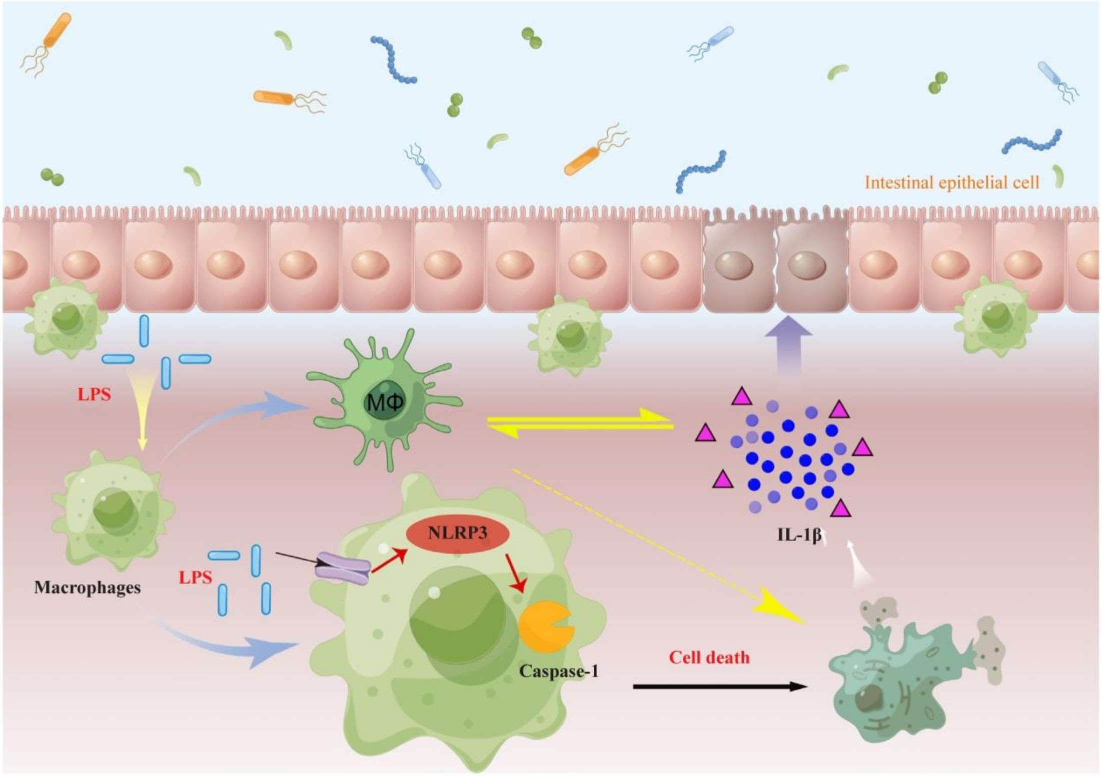

Supplementary Fig. 2 Mechanism of intestinal epithelial cells injury mediated by macrophage pyroptosis (by Figdraw). Infection and other factors trigger the abnormal activation of NLRP3 in intestinal macrophages, which mediates the pyroptosis of macrophages, secretion of inflammatory mediator and the following intestinal epithelial cell injury. *NLRP3* nucleotide-binding oligomerization domain, leucine-rich repeat, and pyrin domain-containing 3, *LPS* lipopolysaccharide, *IL* interleukin
